# Incorporating Topic Assignment Constraint and Topic Correlation Limitation into Clinical Goal Discovering for Clinical Pathway Mining

**DOI:** 10.1155/2017/5208072

**Published:** 2017-05-22

**Authors:** Xiao Xu, Tao Jin, Zhijie Wei, Jianmin Wang

**Affiliations:** School of Software, Tsinghua University, Beijing, China

## Abstract

Clinical pathways are widely used around the world for providing quality medical treatment and controlling healthcare cost. However, the expert-designed clinical pathways can hardly deal with the variances among hospitals and patients. It calls for more dynamic and adaptive process, which is derived from various clinical data. Topic-based clinical pathway mining is an effective approach to discover a concise process model. Through this approach, the latent topics found by latent Dirichlet allocation (LDA) represent the clinical goals. And process mining methods are used to extract the temporal relations between these topics. However, the topic quality is usually not desirable due to the low performance of the LDA in clinical data. In this paper, we incorporate topic assignment constraint and topic correlation limitation into the LDA to enhance the ability of discovering high-quality topics. Two real-world datasets are used to evaluate the proposed method. The results show that the topics discovered by our method are with higher coherence, informativeness, and coverage than the original LDA. These quality topics are suitable to represent the clinical goals. Also, we illustrate that our method is effective in generating a comprehensive topic-based clinical pathway model.

## 1. Introduction

Clinical pathway (CP) is of great importance in disease treatment, hospital management, and government regulation. It has been widely used to make the treatment process more standardized and normalized. The core principle of CP is to divide the clinical treatment into several stages and detail the clinical activities in each stage. Now, there are more than 300 CPs which have been designed by experts and implemented in China.  Table 1 is a Chinese CP example about intracerebral hemorrhage (ICH). However, these CPs can be hardly put into practical application due to their static and nonadaptive feature.

Recently, clinical pathway mining (CPM) has experienced increased attention, which refers to using data mining technologies to discover the execution CP from hospital historical data. In comparison with the expert-designed CP, the data-driven execution CP is more objective. It can facilitate the CP (re)design, CP implementation, and abnormal detection.

The most current CPM approaches are based on the process mining technologies, which are popular in extracting process models from event logs. The resultant graph models contain two basic elements: nodes represent clinical activities and edges represent the order relations between these activities. However, the clinical data are too unstructured and complex for process mining methods. They usually result in spaghetti-like models which are comprised by massive nodes and edges. To make the process model more comprehensive, researchers attempt to manually predefine the key activities for process mining. It is effective in promoting the model conciseness and highlighting core information, while the manual works are always expensive and subjective.

Some other approaches focus on using topic modeling methods to discover clinical patterns instead of process models. They assume that the treatment for a disease can be concluded into a number of patterns. For a patient, a doctor would choose one pattern for him/her according to the symptoms. To achieve this target, topic modeling technologies, such as latent Dirichlet allocation (LDA) [1] and its variants, are applied on the clinical data [2–5]. They model each patient trace as mixtures of multiple topics, while each topic is modeled as a multinomial distribution over clinical activities. However, these approaches can hardly reflect the temporal structure between clinical activities, which are important for CP.

In our previous work [6], we take the advantages of both topic modeling and process mining to generate a concise and interpretable topic-based process model. The key principle is to use LDA to discover the latent topics from the clinical data and extract the order relations between these topics by process mining methods. It is suitable to regard the latent topics as the clinical goals based on the following two clinical practices:

1. The clinical activities occurring in a specific time duration (usually a hospitalized day) are prescribed around several clinical goals.
2. Each clinical goal corresponds to a clinical activity set.

Take the first day of ICH as the example (as shown in Table 1). For a new patient, the doctors would make a diagnosis for him/her by evaluating the intracranial pressure (ICP) and try to control the ICP into the normal range. These constitute the clinical goals for the first day. To achieve these goals, doctors have a series of corresponded choices. Brain CT and the related medical imaging are suitable for the former goal. Dehydration drugs like mannitol and glycerin fructose are very useful for the latter goal. Thus, each clinical day would be represented as several topics instead of the detailed clinical activities. Moreover, the process mining method is effective in discovering the temporal structure in these high-level sequences.

However, the raw LDA can hardly ensure the quality of the discovered topics, which is critical to the interpretability of the final process model. By carefully analyzing the clinical scenario, we find two common problems in applying raw LDA on clinical data.

1. The same clinical activities occurring in one clinical day may be assigned different topics.
2. A clinical activity may have strong correlation to many topics.

**Figure 1:**
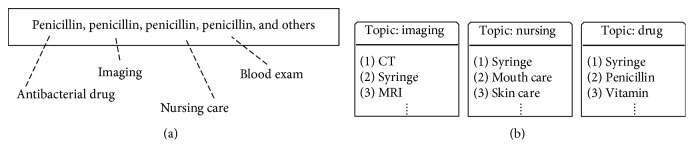
Two problems in applying LDA on clinical data: (a) the clinical activities in one day for a patient and (b) the three discovered topics.

We demonstrate two examples to explain them: (1) In Figure 1(a), the four penicillin prescribed by the doctor in one clinical day are assigned to four different topics; while in the clinical scenario, they are both used for the same clinical goal, which means that they should share the same topic. (2) In Figure 1(b), the clinical activity syringe ranks high in all the three discovered topics. However, each clinical activity should have relative limited number clinical goals rather than various clinical goals.

**Figure 2:**
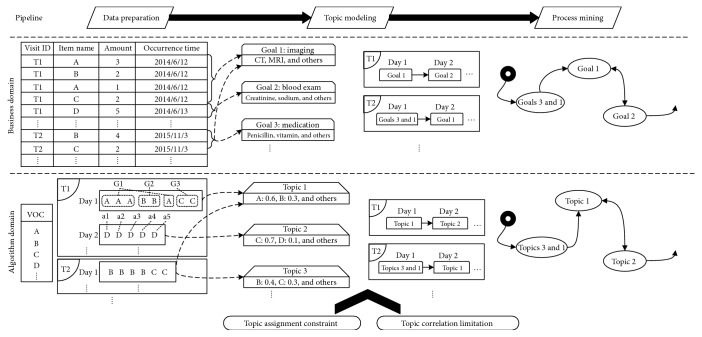
The illustrative process of our approach. The top part is the pipeline, middle part is the business domain, and bottom part is the corresponding algorithm domain (T: patient trace; a: clinical activity; day: clinical day; G: group; VOC: vocabulary).

To address the above problems, we proposed a novel extension of LDA for CPM, which is called Clinical Daily Goal LDA (CDG-LDA). As shown in Figure 2, we first introduce a constraint into LDA which can ensure the same clinical activity in one clinical day would be assigned to the same topic (topic assignment constraint). It can improve the consistency of the topic assignment procedure for clinical goal discovering. Second, we present an efficient method to adaptively limit the topic number of each clinical activity and adjust its ranking in the topic according to their informativeness (topic correlation limitation). It guarantees that a clinical activity would only rank high in limited clinical topics. Third, an effective process mining framework is applied on the topic-based sequences to get a comprehensive process model. It is worth mentioning that we use the billing data as our experimental data. Compared to other various kinds of clinical data, billing data is the most common and credible one due to its importance in insurance area. And it can be easily extracted from hospital information system without complex data integration technologies. Billing data is also of the lowest data granularity and contains a lot of noise, which brings a great difficulty for CPM task. Thus, the CPM approach on the billing data is both valuable and challenged.

Our main contributions in this paper are as follows:

- We incorporate topic assignment constraint into LDA to make the inference procedure consistent for the same clinical activities in one clinical day.
- Informativeness is introduced to adjust the ranking in each topic, which can improve the topic quality.
- Experiments on real-world datasets demonstrate the great performance of our approach for CPM by applying process mining on the quality discovered topics.

We first give the definitions used in this paper:

**Definition 1 (clinical activity).**

A clinical activity is an event occurring at a particular time point, which refers to the basic element in treatment.

**Definition 2 (clinical day and patient trace).**

A patient trace contains a series of clinical activities. We segment each patient trace into a collection of clinical days, and a clinical day represents a set of clinical activities occurring in the same hospitalized day.

## 2. Related Work

Early in 2001, Lin et al. [7] pointed out the shortage of the expert-designed CPs in handling the great variances caused by individual differences. So that a more dynamic and adaptive process is needed for improving the performance of CPs. They proposed a graph mining technique to extract the time dependency pattern of CPs for curing brain stroke. The pattern can be used for predicting the paths for a new patient. In [8], hidden Markov model (HMM) was adopted for inferring the CP pattern, which was useful in knowledge sharing and CP updating. While these methods were severely limited to the data deficiency and complexity and because CPs focus on the order relations between various clinical events, process mining technologies are widely used for CPM. A case study on a group of 627 gynecological oncology patients was presented in [9] by using heuristics miner [10], which resulted in a spaghetti-like process model. Lang et al. [11] evaluated the performance of seven traditional process mining methods on clinical data. The results demonstrated that these methods can hardly obtain comprehensive process models on clinical scenario.

Researchers attempted to adopt frequency-based methods to improve the ability of analyzing the complex process models. In [12], the patient traces with similar schemas and outcomes are grouped together. In [13], sequence clustering was used to identify regular behavior, process variants, and infrequent behavior on the process model discovered by process mining method. Zhang et al. [14] proposed a frequency-based method to group the patients into several predefined core dimensions. Lakshmanan et al. [15] presented a CPM framework that combined clustering, process mining, and frequent pattern mining technologies to mine an outcome-related process model. In [16], a segmentation method was proposed to discover the frequent behavior patterns with different time intervals. CareExplorer [17] was a novel CP management tool which combined frequent sequence mining techniques with advanced visualization supports. Manually defining a set of concerned and important clinical activities can significantly improve the interpretability of the process models [18–20]. However, manual works are always subjective and expensive for CPM.

Considering the complexity and quantity of clinical data, topic modeling technologies are used to discover the clinical patterns, which are useful in CP (re)design and abnormal detection. Huang et al. [2] firstly introduced LDA to extract latent topics as the treatment patterns from clinical data. Each patient trace was regarded as a mixture of different treatment patterns. In addition, the team integrated time stamps [3], patient features [4], and comorbidities [5] into LDA to enhance the ability of summarizing patterns. However, treating a patient trace as a whole can hardly represent the feature of CP that the treatment process is consisted of several stages with different clinical goals. To deal with this problem, our previous work [6] puts the emphasis on discovering the clinical goals from clinical data by LDA and deriving the order relations between these goals by process mining method. It was effective in generating a concise, ordinal, and interpretable process model as CP. While as discussed in [21], the performance of this approach is quite limited to the effectiveness of LDA.

In this paper, we extend the work in [21] to enhance the ability of discovering quality clinical goals by incorporating the topic assignment constraint and topic correlation limitation into LDA.

## 3. Methodology

We first introduce the conversion from billing data to the word-count format for LDA. Then, we present a brief review of applying LDA on clinical data. After analyzing the two shortages of the raw LDA, we incorporate topic assignment constraint and topic correlation limitation into LDA to improve the performance of discovering latent topics from clinical data.

### 3.1. Data Preparation

Billing data is the most common data recorded in hospital information system (an example is shown in the upper-left part of Figure 2). Four basic attributions are essential for the topic modeling: trace ID (the identifier for one patient trace), item name, amount, and occurrence time (in the unit of day). Each billing item (one row in the table) represents a collection of clinical activities, such as the first row <A, 3> means there are three clinical activity A. Note that the amount of different kinds of items should be normalized into the same scale first. We denote the clinical activity domain as the vocabulary. The billing items with the same trace ID and occurrence time constitute one clinical day. With respect to LDA, the clinical day with clinical activities corresponds to the document with words.

### 3.2. Brief Introduction for LDA

**Figure 3:**
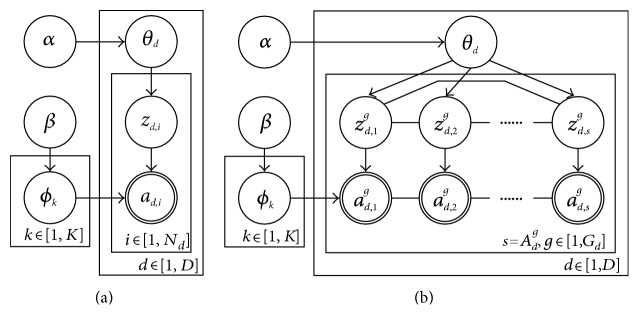
Graphical representation of (a) LDA and (b) CDG-LDA.

LDA is one of the most popular statistical topic modeling technologies. It models the generative process of each word from each document in a text dataset. There are two core model parameters in LDA: a corpus-level distribution over words for each topic and a document-level distribution over topics for each document. In clinical data, by regarding the clinical day and clinical activity as the document and word, respectively, the generative process of LDA has great conformity with the two abovementioned clinical practices. The graphical representation is shown in Figure 3(a), and the notations are explained in Table 2. The generative process of LDA is as follows:

1. Draw distribution *ϕ*_*k*_ ~ Dir(*β*), *k* = 1, 2,…, *K*
2. For *d*th clinical day, *d* = 1, 2,…, *D*
  - Draw distribution *θ*_*d*_ ~ Dir(*α*)
  - For *i*th clinical activity in *d*th clinical day, *i* = 1, 2,…, *N*_*d*_
    - Draw *z*_*d*,*i*_ ~ Multi(*θ*_*d*_)
    - Draw *a*_*d*,*i*_ ~ Multi(*ϕ*_*z*_*d*,*i*__)

According to the graphical model, we need an inference to find the parameters which can best match the observed clinical data. Gibbs sampling, a special case of Markov chain Monte Carlo (MCMC), is the most popular inference method for LDA. It is based on the joint distribution of latent topics and observed clinical activities as follows:

(1) $$\begin{array}{c} P\left(\left.Z,A\right|\alpha ,\beta \right)=P\left(\left.Z\right|\alpha \right)\cdot P\left(\left.A\right|Z,\beta \right)=\int P\left(\left.Z\right|\theta \right)P\left(\left.\theta \right|\alpha \right)d\theta \cdot \int P\left(\left.A\right|Z,\phi \right)P\left(\left.\phi \right|\beta \right)d\phi \propto \overset{K}{\underset{k=1}{\prod }}\left(\overset{D}{\underset{d=1}{\prod }}\Gamma \left(N_{d}^{\left(k\right)}+\alpha_{k}\right)\cdot \frac{\prod_{v=1}^{V}\Gamma \left(N_{k}^{\left(v\right)}+\beta_{v}\right)}{\Gamma \left(N_{k}+\sum_{v=1}^{V}\beta_{v}\right)}\right), \end{array}$$

where Γ(·) is the gamma function.

Given the joint distribution, the topic assignment for each clinical activity can be written as follows:

(2) $$\begin{array}{c} P\left(z_{d,i}=\left.k\right|Z_{\neg z_{d,i}},A\right)=\frac{P\left(Z,A\right)}{P\left(Z_{\neg z_{d,i}},A\right)}\propto \frac{N_{k,\neg z_{d,i}}^{\left(v\right)}+\beta_{v}}{\sum_{v=1}^{V}N_{k,\neg z_{d,i}}^{\left(v\right)}+\beta_{v}}\cdot \left(N_{d,\neg z_{d,i}}^{\left(k\right)}+\alpha_{k}\right). \end{array}$$

After the Gibbs sampling, the two important hidden parameters *ϕ* and *θ* are computed as follows:

(3) $$\begin{array}{c} \begin{array}{c} \phi_{k}^{\left(v\right)}=\frac{N_{k}^{\left(v\right)}+\beta_{v}}{\sum_{v=1}^{V}N_{k}^{\left(v\right)}+\beta_{v}}\\ \theta_{d}^{\left(k\right)}=\frac{N_{d}^{\left(k\right)}+\alpha_{k}}{\sum_{k=1}^{K}N_{d}^{\left(k\right)}+\alpha_{k}}, \end{array} \end{array}$$

where *ϕ*_*k*_^(*v*)^ is the probability of clinical activity *v* which belongs to topic *k* and *θ*_*d*_^(*k*)^ is the probability of the *d*th clinical day which contains topic *k*.

### 3.3. Topic Assignment Constraint

We can see that the inference procedure is mutually independent for all the clinical activities (as shown in (2)). So that even the same clinical activities in one clinical day may get different topics, which violates to the clinical practice.

In this section, we adopt the chain graph proposed in [21] to incorporate topic assignment constraint into LDA. It can coerce the same clinical activities on one clinical day to take on the same topic. The graphical model is shown in Figure 3(b). In each clinical day, we first put all the same clinical activities into a group and then use indirect edges to connect them. A function *C*_*d*_^*g*^ is imposed to express the topic assignment constraint as follows:

(4) $$\begin{array}{c} C\left(z_{d}^{g}\right)=\left\{\begin{array}{ll} 1, & if\;z_{d,1}^{g}=z_{d,2}^{g}=\cdots =z_{d,A_{d}^{g}}^{g}\\ 0, & otherwise. \end{array}\right. \end{array}$$

For each group, *C*_*d*_^*g*^ would equal to 1 only when all the clinical activities in the group get the same topic. Our goal is to make all the groups conform to the constraint. So that we add *C*_*d*_^*g*^ in the joint distribution of LDA (1) as follows:

(5) $$\begin{array}{c} P^{\prime }\left(Z,A\right)=\frac{1}{\Delta }P\left(Z,A\right)\underset{d,g}{\prod }C\left(z_{d}^{g}\right), \end{array}$$

where Δ is the normalization constant.

Similar to the Gibbs sampling algorithm for LDA, we sample a topic for a group based on its posterior *P*′(*z*_*d*_^*g*^ = *k*|*Z*_¬*z*_*d*_^*g*^_, *A*), where *z*_*d*_^*g*^ = *k* represents the set *z*_*d*_^*g*^ which has the same topic *k* (the notations are explained in Table 2).

(6) $$\begin{array}{c} P^{\prime }\left(z_{d}^{g}=k\left|Z_{\neg z_{d}^{g}},A\right.\right)=\frac{P\left(Z,A\right)}{P\left(Z_{\neg z_{d}^{g}},A\right)}\propto \frac{\Gamma \left(N_{d}^{\left(k\right)}+\alpha_{k}\right)}{\Gamma \left(N_{d,\neg z_{d}^{g}}^{\left(k\right)}+\alpha_{k}\right)}\cdot \frac{\Gamma \left(N_{k}^{\left(\alpha_{d}^{g}\right)}+\beta_{\alpha_{d}^{g}}\right)/\Gamma \left(N_{k}+\sum_{v}^{V}\beta_{v}\right)}{\Gamma \left(N_{k,\neg z_{d}^{g}}^{\left(\alpha_{d}^{g}\right)}+\beta_{\alpha_{d}^{g}}\right)/\Gamma \left(N_{k,\neg z_{d}^{g}}+\sum_{v}^{V}\beta_{v}\right)}=\frac{\Gamma \left(N_{d,\neg z_{d}^{g}}^{\left(k\right)}+A_{d}^{g}+\alpha_{k}\right)}{\Gamma \left(N_{d,\neg z_{d}^{g}}^{\left(k\right)}+\alpha_{k}\right)}\cdot \frac{\Gamma \left(N_{k,\neg z_{d}^{g}}^{\left(a_{d}^{g}\right)}+A_{d}^{g}+\beta_{\alpha_{d}^{g}}\right)}{\Gamma \left(N_{k,\neg z_{d}^{g}}^{\left(a_{d}^{g}\right)}+\beta_{\alpha_{d}^{g}}\right)}\cdot \frac{\Gamma \left(N_{k,\neg z_{d}^{g}}+\sum_{v}^{V}\beta_{v}\right)}{\Gamma \left(N_{k,\neg z_{d}^{g}}+A_{d}^{g}+\sum_{v}^{V}\beta_{v}\right)}=\overset{A_{d}^{g}}{\underset{j=1}{\prod }}\left(N_{k,\neg z_{d}^{g}}^{\left(k\right)}+\alpha_{k}+j-1\right)\cdot \frac{N_{k,\neg z_{d}^{g}}^{\left(\alpha_{d}^{g}\right)}+\beta_{\alpha_{d}^{g}}+j-1}{N_{k,\neg z_{d}^{g}}+\sum_{v}^{V}\beta_{v}+j-1}. \end{array}$$

### 3.4. Topic Correlation Limitation

The ranking of a clinical activity *v* in a topic *k* is determined by *ϕ*_*k*_^(*v*)^. From (3), it is observed that *N*_*k*_^(*v*)^ is critical to the calculation of *ϕ*_*k*_^(*v*)^. In addition, the sum of *N*_*k*_^(*v*)^ among all topics is a fixed value, which equals to the frequency of clinical activities *v* (denoted as *N*^(*v*)^) in all clinical days. Due to the independence of the inference procedure of LDA, the frequency of *v* may be uniformly allocated to various topics. Thus, a high-frequency clinical activity is much easier to rank high in many topics, while a low-frequency clinical activity can hardly rank high in even one topic. It results in a large redundancy between different topics, so that each topic is lacking of characteristics. It is hard to regard these topics as the clinical goals.

We consider using informativeness feature to solve this problem. Our insight is that for an informative clinical activity, it should rank high in a small number of topics, which means that it can better represent these clinical goals than other uninformative clinical activities. We incorporate topic correlation limitation into LDA to achieve this target. This limitation contains two aspects:

1. Restrict the distribution of *N*^(*v*)^ over topics. Higher informativeness and less relevant topics
2. Associate the calculation of *ϕ*_*k*_^(*v*)^ with the informativeness of *v*.

We use inverse document frequency (IDF, see (7)) to measure the informativeness of each clinical activity. In general, informative clinical activities are expected to have higher IDF and hence less relevant topics and higher ranking.

(7) $$\begin{array}{c} IDF\left(v\right)=log\frac{D}{\left|\left\{d\in \left[1,D\right]:v\in D_{d}\right\}\right|}. \end{array}$$

Accordingly, the new calculation of *ϕ*_*k*_^(*v*)^ is denoted as follows:

(8) $$\begin{array}{c} {\overset{¯}{\phi }}_{k}^{\left(v\right)}=\phi_{k}^{\left(v\right)}\;\cdot \;IDF\left(v\right). \end{array}$$

The core principle for the first aspect is to keep discarding the most irrelevant topic for a clinical activity. It is achieved by maintaining a relevant topic list for each clinical activity during the iteration of Gibbs sampling. The pseudocode for this procedure is shown in Algorithm 1.

The topic number upper bounder refers to the maximum number of the topics a clinical activity can correlate to. For example, given a clinical activity ECG and its topic number upper bounder *s*(ECG) = 3, its *N*^(*v*)^ can be only allocated to three topics. We set *s*(*v*) from 1 to *K* according to its IDF. Then, for each clinical activity value *v*, we maintain a corresponding relevant topic list *κ*(*v*). In every *δ* iteration, we check whether the size of *κ*(*v*) has been reduced to the desired number *s*(*v*) (line 12). To the clinical activities which still need to be restricted, we will update their lists (line 13) by discarding the topic with minimum relevant value (RV) as defined in

(9) $$\begin{array}{c} RV\left(k,v\right)=\frac{N_{k}^{\left(v\right)}}{rank\left(k,v\right)}, \end{array}$$

where rank(*k*, *v*) is the ranking of clinical activity *v* in topic *k* based on the multinomial distribution *ϕ*_*k*_. The numerator *N*_*k*_^(*v*)^ reflects the relevant degree of topic *k* to clinical activity *v*. And the denominator rank(*k*, *v*) reflects the importance of clinical activity *v* to topic *k*. Thus, higher RV represent more correlation between the clinical activity and the topic.

By keeping on updating the relevant topic lists, we gradually reduce the dimension of topic selection to the desired range. For instance, suppose there are *K* = 5 topics and a clinical activity ECG with a RV vector {50, 150, 5, 100, 60} and *s*(*ECG*) = 3, the relevant topic list *k*(ECG) would be changed from {1, 2, 3, 4, 5} to {1, 2, 4, 5} in the next updating procedure. As seen in line 15 of Algorithm 1, we do the topic sampling from relevant topic list instead of all the topics. To the preinstance, it means that we would sample a topic from topics 1, 2, 4, and 5 without topic 3 because of its minimum RV.

The complexity of deriving a sample *z*_*d*_^*g*^ is *𝒪*(*K*), where *K* is the number of topics. Therefore, the overall complexity is *𝒪*(*KδGK*) = *𝒪*(*K*^2^*δG*). *δ* is the number of iteration for updating relevant topic list and *G* = ∑_*d*_^*D*^*G*_*d*_. In practice, *K* would not be a large value and *δ* could be treated as a constant, so that *G* is the key factor that contributes to the complexity. It means that the proposed method scales linearly as we increase the total number of groups.

### 3.5. Process Mining

Instead of the detailed and complex clinical activities, we use the discovered topics to represent each clinical day. Hence, each patient can be regarded as a topic-based sequence. We adopt the process mining framework proposed in [6] to derive a concise and interpretable process model.

## 4. Experiments

In this section, we conducted a series of experiments to demonstrate the effectiveness of the proposed methods in discovering quality CP model. We begin with the description of experimental settings.

### 4.1. Experimental Settings

To evaluate both the suitability and generality of our approach, we collected two real-world billing datasets from a city-centre hospital. The two datasets are about two different diseases: an internal disease—intracerebral hemorrhage (ICH), and a surgical disease—inguinal hernia (IH). The detailed statistics are summarized in Table 3. We set the Dirichlet prior to *α* = 1.0 and *β* = 0.01.

The topic number *K* is determined by a trade-off strategy proposed in [6]. It attempt to find a balance between the perplexity of topic modeling and the topic label size. The former one is commonly used in evaluating the topic models' prediction ability. It is defined as the reciprocal geometric mean of the likelihood of the data. Lower perplexity refers to better predictiveness and hence a better *K*, while perplexity is not strongly correlated to human judgement [22]. In our approach, the size of topic label is critical to the conciseness of the final process model. High topic number *K* would significantly reduce the interpretability of the model. Thus, we select the intersection point of the two metrics across various *K* as the optimal topic number (*K* = 8 for ICH and *K* = 5 for IH). The iterations for updating relevant topic list *δ* are set to 300.

### 4.2. Topic Quality

The latent topics discovered by topic modeling are interpreted as the clinical goals in our approach. To evaluate the quality of the topics, we compare our approach (CDG-LDA) against LDA [6] in three aspects: coherence, redundancy, and coverage. Tables 4 and 5 show the clustering results of the two methods. For each topic, we listed the top *N* = 10 ranked clinical activities and asked doctors to label it. The similar topics generated by different methods are in the same row. Note that, given a top size *N*, we defined *𝒯* (total top list) as all the top *N*-ranked clinical activities among *K* topics. For example, *𝒯* refers to the 80 clinical activities for ICH when *N* = 10 and *K* = 8.

#### 4.2.1. Coherence

We expect that the clinical activities in a topic could represent the similar clinical goal. Take topic 5 (the 5th row in Table 5) as the example. It is observed that the result of LDA contains both surgery- and nursing care-related activities. While in the result of CDG-LDA, most of the clinical activities are about surgery. The similar situations can be found in 2nd, 4th, and 5th rows of ICH and 2nd row of IH. It shows that the result of approach has significant higher coherent degree than LDA. This may be due to the high co-occurrence of these different kinds of clinical activities. For example, the nursing care activities are usually used with some medication activities in one clinical day, and they all have high-frequency in patients' treatment. Based on the mutually independent topic inference procedure of LDA, it is possible to assign the same topic to parts of the two kinds of activities in one clinical day, such as 60% of nursing care activities and 50% of medication activities got the same topic, and the other activities got different topics. By introducing the topic assignment constraint, we guarantee that the two kinds of activities in one clinical day would be assigned either the same topic or different topics. It can improve the consistency of the topic assignment, which is more conformed to the clinical practice.

We proposed a user study to quantitatively measure the coherence degree of different methods. Three doctors were trained with a short tutorial and invited to label the top 20 clinical activities of each topic to very relevant (score 2), relevant (score 1), or irrelevant (score 0). The final score is determined by a majority voting strategy. The kappa value, which is used to measure the interrater agreement, is 0.73 for ICH and 0.74 for IH. We used NKQM@N [23] as the metric to measure the coherence degree.

(10) $$\begin{array}{c} NKQM@N=\frac{1}{K}\overset{K}{\underset{k=1}{\sum }}\frac{\sum_{j=1}^{N}score\left(M_{k,j}\right)/\log\left(j+1\right)}{Z_{N}}, \end{array}$$

where *K* is the topic number, *M*_*k*_, _*j*_ is the *j*th clinical activity generated by method *M* for topic *k*, and *Z*_*N*_ is the normalization factor. Higher value of NKQM@N implies better ranking result. As shown in Table 6, the performance of CDG-LDA are remarkably better than LDA across various depth of NKQM.

#### 4.2.2. Redundancy

Given two discovered topics, we expect that they would not look alike, which means that the two topics should contain few same clinical activities. Thus, the redundancy indicator focuses on the distinctive characteristic of each topic. We measure the redundancy (RE, see (11)) by calculating the overlapping degree between all the topics.

(11) $$\begin{array}{c} RE=\sum_{v\in \Omega \left(T\right)}\left(count\left(v\right)-1\right), \end{array}$$

where Ω(*𝒯*) is the unique clinical activities in *𝒯* and count(*v*) is the number of the occurrence of clinical activity *v* in the *𝒯* (top *N* clinical activities per topic, *K* topics). In the previous example in Figure 1(b), the RE value among all the three topics is 2 (suppose *N* > 3). Lower RE represents less redundancy and hence a better clustering result.

**Figure 4:**
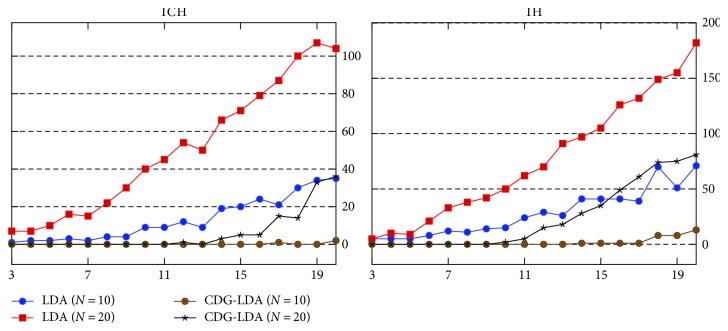
Redundancy indicator RE on various topic number *K* and top size *N*.

Figure 4 shows the comparison between CDG-LDA and LDA. It is observed that CDG-LDA consistently outperforms LDA across various topic number *K* and top size *N*. This is due to the incorporation of topic correlation limitation. By updating the relevant topic list during the iterations of Gibbs sampling, each clinical activity would strongly correlate to limited topics, especially the informative clinical activities. As a sequence, a clinical activity would not rank high in many topics, which lead to a low redundancy degree among all the topics.

#### 4.2.3. Coverage

Coverage refers to the ability of discovering important clinical activities for a specific disease. Given two high coherent and low redundant topics (syringe, infusion apparatus and arterial/venous cannula, hemostix) for ICH, both of them are about the basic clinical equipment. However, these clinical activities are not critical enough for the treatment of ICH. We aim to find out all the key clinical activities in *𝒯*. We used the necessary/recommendatory clinical behaviors in the Chinese National Clinical Pathway and Chinese/AHA/ASA/EHS guideline as our benchmark. The overlapping degree between *𝒯* and the benchmark is adopted as the coverage indicator. Here, we took the *𝒯* listed in Tables 4 and 5, which means the top size *N* = 10, to analyze the coverage from three aspects:

- Examination

For ICH, the core examinations contains blood/urine routine, ECG, and biochemical and imaging examinations. For the first three, both LDA and CDG-LDA have discovered majority of the related clinical activities in topics 5, 6, 7, and 8. While for the last one, LDA failed to find out brain CT, which is the most effective imaging examination for ICH. In CDG-LDA, brain CT ranked high in topic 5.

For IH, a patient needs the similar examinations to ICH except the imaging test. In topics 1 and 3 of the two methods, we can find the clinical activities about blood/urine routine, ECG, and biochemical examinations. An interesting observation is that the topics in the 2nd row got by LDA and CDG-LDA are quite different. In LDA, the activities in topic 1 are mainly about the infectious disease assay, which is an important part of biochemical examination. While topic 2 of CDG-LDA focuses on the presurgical preparation. Although LDA got better performance in infectious disease examinations, we deem that the result of CDG-LDA is more suitable for the clustering of IH clinical behaviors. There are two reasons: (1) The infectious disease assay-related activities can be found in the top 20 of topic 1 in CDG-LDA. They all belonged to the biochemical examinations which will always be prescribed together in practice, so that it is reasonable to put them in the same topic. (2) Topic 2 about presurgical preparation is of great importance for IH. We would detail this in the following part.

(ii) Treatment

Due to the ICH dataset is extracted from the Neurology Department rather than the Neurosurgery Department, the treatment activities are mainly medical instead of surgical. According to the drug function, the commonly used medication for ICH can be divided into four categories: reducing ICP (dehydration), keeping electrolyte balance, controlling blood pressure, and preventing complications (like dysphagia and aspiration, nervous system injury, bacterial infection, etc.). Most of them have been covered by both LDA and CDG-LDA. However, LDA missed an important dehydration drug piracetam, which is of high frequency in the dataset.

IH is a typical surgical disease, so that the treatment activities are mainly about the surgery. It is observed that both LDA and CDG-LDA contained the surgery-related clinical activities in topic 5. However, LDA failed to discover the most critical surgical activity IH repair and anesthesia in the top 10 of topic 5. This is caused by the high redundancy between topics 4 and 5 of the LDA. It made the ranking of these important surgical activities lower than the nursing care activities, which are of high-frequency. Moreover, as we discussed before, few of presurgical activities have been found by LDA. By limiting the topic number of each clinical activity and adjusting the day-activity distribution calculation, CDG-LDA successfully discovered these clinical activities in topic 4.

(iii) Nursing care

In both ICH and IH datasets, the frequency of the clinical activities about nursing care are always the highest. Hence, the two topic modeling methods got good performance in discovering this kind of activities, including different level nursing care and various nursing equipment.

*(1) Discussion of Topic Quality.* From the abovementioned three aspects about coherence, redundancy, and coverage, we can tell that CDG-LDA demonstrates significantly better performance than the original LDA in discovering latent topics from clinical data. The extracted high-quality topics can be suitably interpreted as the clinical goals.

### 4.3. Process Model Demonstration

**Figure 5:**
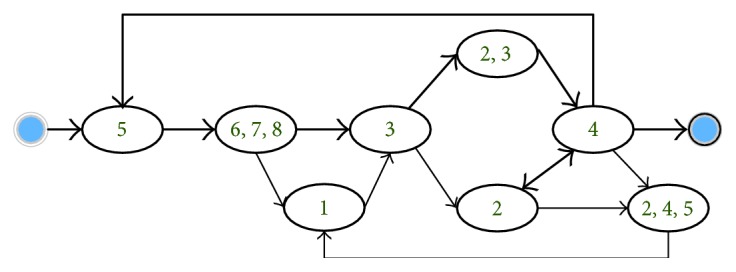
ICH process model.

**Figure 6:**
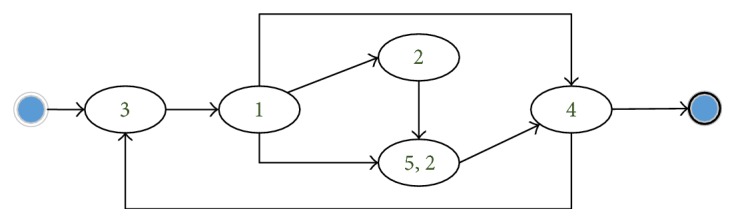
IH process model.

By using a topic label (contains several topics) to represent each clinical day, we got a series of topic-based sequences. In this section, we demonstrate the process models derived from these sequences by using the process mining methods proposed in [6]. Note that we combined the topics about biochemical examinations, which are always used together, for the sake of discussion. Figures 5 and 6 show the result model of ICH and IH, respectively. We compared them with the Chinese National Clinical Pathways.

- ICH

The process can be easily divided into three stages.

1. Admission (topic (5), topic (6, 7, and 8)): the patient would accept a series of examinations for making a definite diagnosis, including brain CT, ECG, and biochemical assays. Note that imaging examinations are not required for all the traces. This is due to the fact that the clinical activities in our imaging topic (topic (1)), such as CTA and MRA, are mainly used as the auxiliary means for the diagnosis of ICH. Usually, the patients with severe symptoms or comorbidities need further imaging examinations.
2. Treatment (topic (3), topic (2, 3), topic (4), and topic (2)): After various examinations, the ICH patients would receive nursing care and take medications for recovery. Because of the urgency of ICH, drugs and high-level nursing care are firstly used to relieve and monitor the symptoms. When symptoms become stable, the nursing level may be changed to regular. As an internal department, various medications are used for ICH treatment according to the patients' symptoms.
3. Re-examination (topic (5), topic (2, 4, and 5), and topic (1)): During the treatment stage, related examinations are repeatedly needed for confirming the patients' states.
4. IH

Similar to ICH, the IH patients would firstly receive various examinations. Then, a series of presurgical activities are used for them. Note that some patients would have surgery in the same clinical day to the presurgical preparations (topic (5, 2)), while others may accept surgery in the next clinical day (topic (2)). The average level of nursing care for IH is lower than ICH. And the antibacterial drugs are used during the surgery and nursing care period. It is worth mentioning that the majority of IH patients have undergone the surgical treatment; while it still existed, a small number of IH patients have not received surgery. By careful analysis, we found that these patients are mainly the infants or the elders who are not appropriate for surgery.

*(1) Discussion of Process Model*. It is observed that the topic-based clinical process model is of great conciseness and interpretability for CPM. We can conveniently draw the execution CP from the clinical historical data and identify the characteristics compared to expert-designed CP.

## 5. Conclusion

In this paper, we proposed a novel topic modeling approach to discover latent topics as the clinical goals for CPM. The key idea is to incorporate clinical practice into the generative process of LDA to improve the topic quality. Topic assignment constraint restricts that all the same clinical activities in one clinical day should be assigned the same topic. Topic correlation limitation incorporates informativeness feature to adaptively adjust the ranking of the clinical activities in each topic. Process mining methods are used on these topic-based sequences instead of the detailed clinical activities. The experimental results show that our approach outperforms the original LDA in coherence, redundancy, and coverage. And the resultant topic-based process models are of great conciseness and interpretability for CP representation.

One extension of this work is to find a more suitable measure as the informativeness indicator to improve the ranking adjustment strategy.

## Conflicts of Interest

The authors declare that there is no conflict of interest regarding the publication of this paper.

## Figures and Tables

**Algorithm 1:**
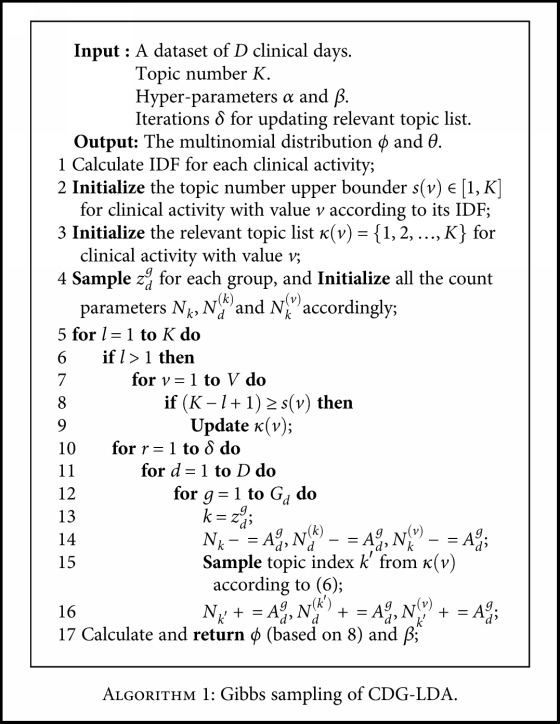
Gibbs sampling of CDG-LDA.

**Table 1:** The national clinical pathway of intracerebral hemorrhage released by the Ministry of Health of China.

Stage	Order
Stage 1 (Day 1)	Long-term medical order:
	(1) neurology nursing routine, (2) level I care, (3) normal diet, (4) keep the bed, and (5) observing vital signs
	Temporary medical order:
	(1) blood, urine, and stool routine examination; (2) hepatorenal function, electrolytes, blood glucose, blood lipids, cardiac enzymes, coagulation function, and infectious disease screening; (3) brain CT, chest X-ray, and ECG; and (4) when necessary: brain MRI, CTA, MRA, or DSA

Stage 2 (Day 2)	Long-term medical order:
	(1) neurology nursing routine, (2) level I care, (3) normal diet, (4) keep the bed, (5) observing vital signs, and (6) basic drugs
	Temporary medical order:
	(1) re-examination for abnormal laboratory values and (2) when necessary: re-examination CT

Stage 3 (Day 3)	Long-term medical order:
	(1) neurology nursing routine, (2) level I care, (3) normal diet, (4) keep the bed, (5) observing vital signs, and (6) basic drugs
	Temporary medical order:
	(1) re-examination for abnormal laboratory values

Stage 4 (Days 4–6)	Long-term medical order:
	(1) neurology nursing routine, (2) level II care, (3) normal diet, (4) keep the bed, (5) observing vital signs, and (6) basic drugs
	Temporary medical order:
	(1) re-examination for abnormal laboratory values; (2) re-examination for blood, kidney function, blood glucose, and electrolytes; and (3) when necessary: re-examination CT

Stage 5 (Days 7–13)	Long-term medical order:
	(1) neurology nursing routine, (2) level II-III care, (3) normal diet, (4) observing vital signs, and (5) basic drugs
	Temporary medical order:
	(1) re-examination for abnormal laboratory values and (2) when necessary: DSA, CTA, and MRA

Stage 6 (Days 8–14)	Long-term medical order:
	(1) discharge with drugs

**Table 2:** Meanings of the notations.

Notation	Meaning
*D*	The set of clinical days
*A*	The set of clinical activities
*Z*	The set of topics assigned to *A*
*D*, *K*	The number of clinical days and topics
*V*	The number of unique clinical activities
*N*_*d*_	The number of clinical activities in *d*th clinical day
*N*_*k*_	The number of clinical activities that are assigned topic *k*
*G*_*d*_	The number of groups (unique clinical activities) in *d*th clinical day
*A*_*d*_^*g*^	The number of clinical activities in *g*th group in *d*th clinical day
*a*_*d*,*i*_	The *i*th clinical activities in *d*th clinical day
*z*_*d*,*i*_	The topic for *a*_*d*,*i*_
*a*_*d*_^*g*^	The clinical activities of *g*th group in *d*th clinical day
*a*_*d*,*j*_^*g*^	The *j*th clinical activity in *g*th group in *d*th clinical day
*z*_*d*,*j*_^*g*^	The topic for *a*_*d*,*j*_^*g*^
*z*_*d*_^*g*^	{*z*_*d*,*j*_^*g*^}_*j*=1_^*A*_*d*_^*g*^^, the set of topics for *g*th group in *d*th clinical day
*N*_*d*_^(*k*)^	The number of clinical activities that are assigned topic *k* in *d*th clinical day
*N*_*k*_^(*v*)^	The number of clinical activities with value *v* and topic *k*
*N*_*d*,¬*z*_*d*_^*g*^_^(*k*)^	The number of clinical activities that are assigned topic *k* in *d*th clinical day, except *g*th group in *d*th clinical day
*N*_*k*,¬*z*_*d*_^*g*^_^(*v*)^	The number of clinical activities with value *v* and topic *k*, except *g*th group in *d*th clinical day
*α*, *β*	Dirichlet prior vector
*ϕ*, *θ*	Multinomial distribution over clinical activities and topics
*ϕ*_*k*_	Multinomial distribution over clinical activities for topic *k*
*θ*_*d*_	Multinomial distribution over topics for *d*th clinical day

**Table 3:** Statistics of our datasets.

Disease	Trace number	Day number	Voc number	Avg LOS	Min LOS	Max LOS
ICH	240	3204	752	14	2	34
IH	33	241	447	6	2	10

**Table 4:** Eight labeled topics of ICH by different methods.

Number	Topic tag	LDA	Topic tag	CDG-LDA
1	Imaging	(1) Doppler echocardiography, (2) Doppler ultrasonography of left ventricular function, (3) ventricular wall motion, (4) transcranial Doppler, (5) analysis of ultrasonic, (6) color Doppler ultrasonography of abdomen, (7) X-radiography, (8) digitized photography, (9) B mode ultrasound, and (10) film	Imaging	(1) Doppler echocardiography, (2) Doppler ultrasonography of left ventricular function, (3) ventricular wall motion, (4) transcranial Doppler, (5) analysis of ultrasonic, (6) color Doppler ultrasonography of the abdomen, (7) X-radiography, (8) B mode ultrasound, (9) digitized photography, and (10) film

2	Medication	(1) Aceglutamide, (2) sodium chloride, (3) local infiltration anesthesia, (4) etimicin, (5) cerebrosidekinin, (6) physical cooling, (7) t-branch pipe, (8) aerosol inhalation, (9) ambroxol, and (10) venous transfusion	Medication	(1) Pantoprazole, (2) potassium magnesium aspartate, (3) mannitol, (4) vitamin B6, (5) glycerin fructose, (6) vitamin C, (7) naloxone, (8) piracetam, (9) sodium chloride, and (10) glucose

3	High-level nursing care	(1) Level I care, (2) ECG monitoring, (3) blood oxygen saturation monitoring, (4) urinary meatus care, (5) mouth care, (6) skin care, (7) hospital examining fee, (8) mouth care package, (9) ambulatory blood pressure monitoring, and (10) continuous oxygen inhalation	High-level nursing care	(1) Level I care, (2) ECG monitoring, (3) blood oxygen saturation monitoring, (4) mouth care, (5) urinary meatus care, (6) skin care, (7) continuous oxygen inhalation, (8) mouth care package, (9) ambulatory blood pressure monitoring, and (10) drainage pack

4	Regular nursing care and medication	(1) Level II care, (2) hospital examining fee, (3) pantoprazole, (4) vitamin B6, (5) mannitol, (6) vitamin C, (7) potassium magnesium aspartate, (8) venous transfusion, (9) sodium chloride, and (10) glycerin fructose	Regular nursing care	(1) Level II care, (2) venous transfusion, (3) flusher, (4) syringe, (5) arterial/venous cannula, (6) therapy application, (7) aceglutamide, (8) intramuscular injection, (9) physical cooling venipuncture, and (10) nifedipine

5	Admission examination	(1) ECG, (2) PLGA, (3) fibrous protein, (4) blood collection tube, (5) hemostix, (6) washbasin, (7) ECG event log, (8) plasma prothrombin time assay, (9) activated partial thromboplastin time, and (10) prothrombin time assay	Admission examination	(1) Brain CT, (2) venous sampling, (3) ECG, (4) hemostix, (5) electrode, (6) 12 channel dynamic electrocardiogram, (7) washbasin, (8) ECG event log, (9) nasogastric, and (10) evaluation of activities of daily living

6	Biochemical exam	(1) Urea assay, (2) creatinine assay, (3) uric acid assay, (4) chloride assay, (5) blood cell analysis, (6) potassium assay, (7) sodium assay, (8) glucose assay, (9) serum bicarbonate assay, and (10) excrement	Biochemical exam	(1) Creatinine assay, (2) urea assay, (3) uric acid assay, (4) blood cell analysis, (5) chloride assay, (6) sodium assay, (7) potassium assay, (8) serum bicarbonate assay, (9) glucose assay, and (10) calcium assay

7	Biochemical exam	(1) Serum a-L-glucosidase assay, (2) serum 5′ nucleotidase assay, (3) plasma viscosity assay, (4) glycosylated hemoglobin assay, (5) whole blood viscosity, (6) identification of Rh blood group antigen, (7) amylase assay, (8) serum creatine kinase-MB isoenzyme activity assay, (9) lactate dehydrogenase assay, and (10) serum alanine aminotransferase assay	Biochemical exam	(1) Serum 5′ nucleotidase assay, (2) serum a-L-glucosidase assay, (3) plasma viscosity assay, (4) serum creatine kinase-MB isoenzyme activity assay, (5) glycosylated hemoglobin assay, (6) whole blood viscosity, (7) identification of Rh blood group antigen, (8) amylase assay, (9) lactate dehydrogenase assay, and (10) serum total protein assay

8	Biochemical exam	(1) Serum aspartate aminotransferase assay, (2) electrophoresis analysis of lactate dehydrogenase isoenzymes, (3) urine analysis, (4) serum 7 pancreatic acyltransferase assay, (5) serum alkaline phosphatase assay, (6) serum alanine aminotransferase assay, (7) urinalysis, (8) hepatitis A antibody assay, (9) serum high-density lipoprotein cholesterol assay, and (10) serum total cholesterol assay	Biochemical exam	(1) Serum aspartate aminotransferase assay, (2) serum albumin assay, (3) urine sediment quantitative analyze, (4) inorganic phosphorus assay, (5) electrophoresis analysis of lactate dehydrogenase isoenzymes, (6) serum fructose amine assay, (7) urine analysis, (8) serum 7 pancreatic acyltransferase assay, (9) serum alanine aminotransferase assay, and (10) serum alkaline phosphatase assay

**Table 5:** Five labeled topics of IH by different methods.

Number	Topic tag	LDA	Topic tag	CDG-LDA
1	Biochemical exam	(1) Blood cell analysis, (2) glucose assay, (3) creatinine assay, (4) urea assay, (5) chloride assay, (6) sodium assay, (7) potassium assay, (8) uric acid assay, (9) calcium assay, and (10) magnesium assay	Biochemical exam	(1) Blood cell analysis, (2) glucose assay, (3) sodium assay, (4) chloride assay, (5) potassium assay, (6) uric acid assay, (7) creatinine assay, (8) urea assay, (9) calcium assay, and (10) magnesium assay

2	Infectious disease exam	(1) Hepatitis A antibody assay, (2) treponema pallidum antibody assay, (3) HIV antibody assay, (4) hepatitis B core antibody assay, (5) hepatitis Be antibody assay, (6) hepatitis C antibody assay, (7) hepatitis B surface antibody assay, (8) hepatitis B surface antigen assay, (9) urine analysis, and (10) urinary sediment microscopy	Presurgical preparation	(1) Skin preparation package, (2) skin preparation, (3) disinfection fee, (4) hygienic material, (5) washbasin, (6) intramuscular injection, (7) health education, (8) BP monitoring, (9) cefuroxime, and (10) infusion apparatus

3	Admission exam	(1) ECG, (2) ECG monitoring, (3) ABO subtype assay, (4) urine routines, (5) prothrombin time assay, (6) activated partial thromboplastin time, (7) plasma prothrombin time assay, (8) washbasin, (9) Rh subtype assay, and (10) color Doppler ultrasonography of the abdomen	Admission exam	(1) ECG, (2) ECG monitoring, (3) urine routines, (4) urinary sediment microscopy, (5) ECG event log, (6) abdomen X-ray, (7) chest X-ray, (8) color Doppler ultrasonography of superficial organ, (9) color Doppler ultrasonography of abdomen, and (10) analysis of ultrasonic

4	Regular nursing care	(1) Level II care, (2) hospital examining fee, (3) level III care, (4) infusion apparatus, (5) venous transfusion, (6) hemostix, (7) syringe, (8) special hemostix, (9) dressing change, and (10) arterial/venous cannula	Regular nursing care	(1) Level II care, (2) level III care, (3) dressing change, (4) fat-soluble vitamin, (5) cefotiam, (6) arterial/venous cannula, (7) venous transfusion, (8) small dressing change, (9) levofloxacin, and (10) middle dressing change

5	Surgery and regular nursing care	(1) Level II care, (2) endotherm knife, (3) mask, (4) venous transfusion, (5) hospital examining fee, (6) glucose, (7) syringe, (8) ECG monitoring, (9) blood oxygen saturation, and (10) sodium chloride	Surgery	(1) Mask, (2) endotherm knife, (3) surgery package, (4) blood oxygen saturation, (5) lumbar anesthesia, (6) epidural anesthesia, (7) application, (8) oxygen therapy, (9) IH repair, and (10) anesthesia monitoring

**Table 6:** Topic coherence in terms of NKQM@N.

Dataset	Method	NKQM@5	NKQM@10	NKQM@20
ICH	LDA	0.8211	0.8004	0.7907
	CDG-LDA	0.8448	0.8498	0.8235

IH	LDA	0.7915	0.7830	0.7631
	CDG-LDA	0.8316	0.8266	0.8116
